# South Korea: in the midst of a privacy reform centered on data sharing

**DOI:** 10.1007/s00439-018-1920-1

**Published:** 2018-08-18

**Authors:** Hannah Kim, So Yoon Kim, Yann Joly

**Affiliations:** 1 Asian Institute for Bioethics and Health Law, Yonsei University, Seoul, South Korea; 2 Centre of Genomics and Policy, McGill University, Montreal, Canada

## Abstract

With rapid developments in genomic and digital technologies, genomic data sharing has become a key issue for the achievement of precision medicine in South Korea. The legal and administrative framework for data sharing and protection in this country is currently under intense scrutiny from national and international stakeholders. Policymakers are assessing the relevance of specific restrictions in national laws and guidelines for better alignment with international approaches. This manuscript will consider key issues in international genome data sharing in South Korea, including consent, privacy, security measures, compatible adequacy and oversight, and map out an approach to genomic data sharing that recognizes the importance of patient engagement and responsible use of data in South Korea.

## Introduction

With rapid developments in genomic and digital technologies, genomic data sharing has become key to achieving precision medicine (Knoppers et al. 2014; National Research Council 2011). One of the main shifts comes from the field of epigenetics that offers new opportunities for genomic research, disease prevention (Perkins et al. 2018) and precision medicine (Grossman et al. 2016). Following on these advances, South Korea made data sharing for precision medicine a priority in its political agenda (Korea Institute of S & T Evaluation and Planning 2017). Two decades ago, the initial biobanks in South Korea were population-based cohort studies. Initiated in 2008, the Korea Biobank Network (KBN) now includes 17 regional biobanks, and the National Biobank of Korea (NBK) systemically collects human bioresources. The NBK has collected, managed and distributed human biospecimens and data through the Biospecimen Information Management System (BIMS) (Korea National Institute of Health 2017). Today, the leading agency of the KBN and a control center of the NBK is the Korea Centers for Disease Control and Prevention (KCDC). It is currently planning a large-scale precision medicine databank (Chu 2017; Korea Institute of S & T Evaluation and Planning 2017).

Various genomic data sharing policies exist at the international level (Global Alliance for Genomics and Health 2014; OECD 2007; UNESCO 2003). South Korea now also stands in a position to utilize its abundant national data resources in healthcare and join international research collaborations. Clinical data are deposited in the electronic medical records (EMRs) in 92% of hospitals (Korea Ministry of Health and Welfare 2016) and the records of hospitalization and medical claims are transmitted to the National Health Insurance Corporation and the Health Insurance Review and Assessment Service. Moreover, there is a multi-ministerial effort to integrate public health data with other public data, such as geospatial data collected by the Ministry of Land, Infrastructure and Transportation or Statistics Korea; environmental and satellite data from the Meteorological Administration or from the Ministry of Environment; population census, household income and expenditure survey data from Statistics Korea; as well as birth- and death-related data from the Ministry of the Interior and Safety (Kim et al. 2017; Gang 2016). Through this national initiative, the government aims to enable high accessibility and use of data among South Korean researchers (Chu 2017) in a similar fashion as the UK Biobank (Sudlow et al. 2015), the Canadian Partnership for Tomorrow Project (Drummer et al. 2018) and the All of Us research program in the U.S. (Devaney 2017).

There are also growing interconnections between the public and commercial domains associated with precision medicine. Since 2016, South Korea has permitted genetic testing outside of the clinical setting, including private Direct-to-Consumer Genetic Testing (DTC-GT) (Jeong 2017). DTC-GT regulations in the revision of the *Bioethics and Safety Act* of 2005 (BSA) (2017) apply to commercial genomic data sharing and public–private partnerships. Major hospitals also collaborate to develop their own big data for clinical research. For example, in 2015 the Asan Medical Center, one of the major private hospitals in South Korea, developed a research information system with more than 4 million registered patients that present a first model of big data research in hospitals (Shin et al. 2015).

The integration of real-life data generated from personal mobile and wearable devices to conduct research will enrich precision medicine. Patients and citizens can be considered as data providers. In that sense, individuals can share life-logs and biometric information such as diet, sleep, physical activity and movement to any database through digital technologies. Given the potential of these new tools, active public engagement is becoming a crucial component to successfully increase participation in genomic research (Borry et al. 2018). Moreover, there is substantial evidence that active public engagement and sharing personal life data are overall beneficial to the individuals as digital health consumers (McMahon and Pan 2018; Parker et al. 2018; Willink and DuGoff 2018; Rathbone and Prescott 2017). Thus, the success of data-intensive models for precision medicine relies largely on public trust and positive anticipation of research benefits.

**Fig. 1:**
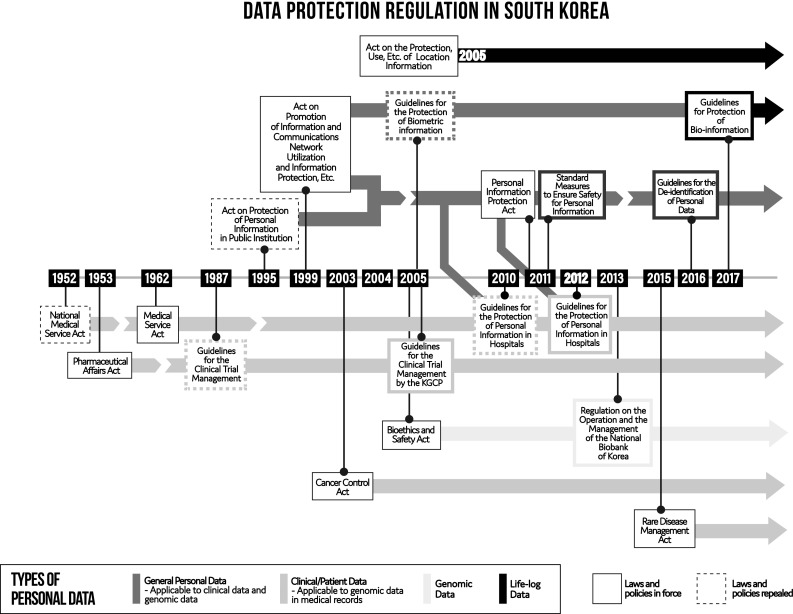
Data protection regulation in South Korea

Emerging international trends in data-sharing initiatives have brought new opportunities along with ethical, legal and political challenges (Robinson et al. 2015). Data can be potentially re-identified, which has led to illicit data misuse cases (Phillips et al. 2017). Personal information stored in a database cannot be destroyed if it has been copied or shared with others. Indeed, there are concerns associated with data governance and data sharing, such as privacy, security and liability, as well as commercial use (Allen et al. 2014) raising the need to review Korean data governance policies regarding the different size, scope, complexity, scale and forms of genomic data sharing on the horizon. Accordingly, the South Korean government has consolidated its data protection legislation which will also apply to international genomic data sharing (Fig. 1). Despite these legislative changes, the BSA and the *Personal Information Protection Act* of 2011 (PIPA) (2017) remain relevant and applicable. This manuscript considers key issues in international genomic data sharing in South Korea, including consent, privacy, security measures, compatible adequacy and oversight. It maps out an approach to genomic data sharing that recognizes the importance of both patient engagement and the responsible use of genomic data in South Korea.

## Consent

The PIPA is a general law regarding the processing and protection of personal data (Art 1). According to the PIPA, a data subject is defined as “an individual who is identifiable by the information processed hereby becomes the subject of that information” (Art 2-3) and any party that processes personal information directly or indirectly to utilize the personal information is defined as a personal information controller, such as public institutions, corporations and a data user or a researcher (Art 2-5).

With regard to consent, a personal information controller bears certain responsibilities under the PIPA. A data controller should obtain the data subject’s consent to the collection and use of personal information (Art 15). The PIPA requires that a data subject providing her/his personal information be notified by the data controller regarding the purpose of the collection and use of this information, the type of personal information being collected, the period of holding and usage of the personal information, and the right to refuse or withdraw consent. The data-sharing activities of a personal information controller are limited by the PIPA. A personal information controller must collect and identify the minimum personal information necessary to achieve the purpose of the collection (Art 16-1). The controller should then inform the data subject of the minimum personal information that is required so that the data subject may withhold her/his consent regarding the collection of information beyond this minimal dataset (Art 16-2). Additionally, the party collecting the information cannot refuse to provide goods or services to a person if he/she does not agree with the provision of information other than the required information (Art 16-3). However, it is challenging to clearly distinguish what constitutes the minimum information required from information that goes beyond the required information. Unfortunately, the PIPA does not provide additional guidance on this distinction. In other words, according to the PIPA, it is unclear which is the personal information that can be freely used by corporations and public institutions and which is the personal information that can only be used by obtaining the specific consent of data providers.

These principles apply consistently, but with different criteria, including the possibility for broad consent for biobanks and genomic researches, to parties collecting genetic resources under the BSA. In a biobank context, it is mandatory to obtain written consent from the biospecimen donor and the range of researchers and institutions party to the data sharing must be indicated in the consent form (Art 42-1-3). After a biobank collects biospecimens with such consent, no further consent is necessary for further genomic data sharing. The biobank is responsible for reviewing the research proposals submitted by third parties and decides whether to provide biospecimens or genomic data (Art 43-1). If a genomic researcher plans to collect biospecimens or genomic data directly from research participants, written consent should be obtained along with information plans concerning genomic data sharing (Art 37-1-4). The research proposal, including the consent form and a plan for genomic data sharing, should also be approved by an Institutional Bioethics Committee (IBC) (Art 36-1) and the researcher can only provide them to a biobank or to other researchers after receiving this approval (Art 38-1). E-consent is accepted as an alternative to written consent under the BSA (Art 16-1). For biobank projects, the specific purpose of genomic research only needs to be mentioned in the consent form when a biobank is established to carry out a specific genomic research project, such as research on pulmonary cancer or leukemia (Art 42-1-1). Generally, biobanks seek consent for further data and biospecimen use without mentioning a specific research purpose (BSA Enforcement Ordinance, Annex 41). This broad consent model is now acceptable under the BSA (Kim et al. 2017; You and Kim 2015).

Although broad consent is generally used in biobanking, there are some challenges when applying this type of consent to biobanking for precision medicine. Foremost, legal guidelines on consent to use collected clinical data and biological samples which are a part of medical care records are unclear. Article 21-2 in the *Medical Service Act* of 1962 (MSA) (2017) does not allow the release of a patient’s medical record to a third party, including his/her spouse, lineal ascendant or descendant or sibling, by a medical provider, the head of a hospital or a person working at a hospital. This provision is applied to prevent any disclosure or transmission of a patient’s data for research purposes to a third party without proper consent. Researchers can use clinical data only if they satisfy the requirements of consent present in both the PIPA and the BSA. According to the PIPA, a hospital can provide clinical data to a third party when patients have consented to provide the data (Art 17-1, 2) and an additional separate consent is needed for cross-border data sharing (Art 17-3). A researcher can also share anonymized clinical data with the research community if the initial research plan is approved by the IBC and if the patient agreed to this sharing on the initial consent form (BSA 18-1, 2). When the data are shared, a second approval by the IBC is needed. A hospital, as a personal information provider, can provide clinical data without consent only if the data are de-identifiable and used for statistics or academic research (Art 18-2-4). Although there is no further explanation provided in the BSA as to whether de-identification is the same concept as that of anonymization (Art 38-2), specific information on de-identification and anonymization is only provided in a governmental guideline (Office for Government Policy Coordination et al. 2016). The lack of certainty around this definition could mean that, even if a hospital follows the governmental guideline, it could still be found liable in case of a breach of the duty of confidentiality (Kwon et al. 2016).

There are ongoing discussions on the opportunity to provide consent exemptions to research using personal health data from public health databases. The major argument to support a consent exemption is that it is necessary to link personal data from public and private databases in precision medicine, but this type of linkage is impossible if the data research has to satisfy the PIPA and the BSA because this requires data to be de-identified which, in this context, should be interpreted as anonymized (Kwon et al. 2016; Korea Institute of S & T Evaluation and Planning 2017). The Cancer Registration Statistics Service provides an example of a database where such an exemption of consent already exists. The Ministry of Health and Welfare has the authority for the registration of cancer patients, the management of the National Cancer Registry database and research through continuous and systematic collection and analysis of data without consent. Thus, the PIPA’s consent requirement does not apply to research authorized by the National Cancer Center under the *Cancer Control Act* of 2003 (2017) (Art 14). A similar consent exemption applies to data collected from rare disease patients in the National project for registration and statistics for rare disorders under the *Rare Disease Management Act* of 2016 (2016) (Art 10-1).

There is also a discussion on the suitability of a consent exemption for the usage of personal location information transmitted from mobile devices. In the *Act on the Protection, Use, Etc. of Location Information* of 2005 (2017), personal location information is defined as “the location information regarding a particular person including information combinable with other information to track the location of a particular person even though location information alone is not sufficient to identify the location of such person” (Art 2-2). Similar to the All of Us program in the U.S. (Rothstein 2017; Devaney 2017), the precision medicine database in South Korea plans to collect personal mobility data (Korea Institute of S & T Evaluation and Planning 2017). In principle, the Act requires that any location-based service provider obtain consent that indicates the designated recipient of the personal location information and the purpose of sharing this data with a third party (Art 19). Currently, consent is exempt where data are de-identified and provided for the purpose of statistics, academic research or market research to the third party by a location information provider, such as mobile carrier companies or mobile applications (Art 21-2). However, this seems illogical, for it is impossible to provide de-identified information in this case since personal location data contain dynamic information about time, place and movement, as well as sensitive information about close relatives, dietary style, religion, education, job, etc. and location data can also be combined with other data in academic research. Moreover, Article 21-2 should not cover this type of usage of combined data because of its potential to be re-identified in such large-scale public datasets (de Montioye et al. 2013; Rodriquez et al. 2013) and the inherent risk of re-identifiability of public biobanks linked with a geo-information system (Bovenberg et al. 2016). Arguably, consent exemption, even for academic research, should not apply and separate consent from the data subject should be required to collect and use location information, particularly because of the contentious use term of the ‘de-identification’ (Phillips and Knoppers 2016).

From those discussions, as an alternative to broad consent or exemption from consent, a dynamic consent process is currently also considered by stakeholders. Current discussions in consent are mainly to seek a balance between the protection of the interests and autonomy of data subjects and the optimization of the sustainability of databases (International Bioethics Committee of the UNESCO 2017; Ploug and Holm 2016; Kaye et al. 2015; Steinsbekk et al. 2013). A main concern with broad consent is that this model would not permit researchers to provide sufficient information about the further usage of individual data to research participants (Grady et al. 2016). Dynamic consent is expected to resolve this problem using IT to facilitate the accomplishment of specific consent objectives with respect to addressing research participants’ autonomy but there are still limitations on this type of consent (Joly 2018).

## Privacy

In the past, South Korea developed separate laws to regulate the use of personal data in the public and private sectors. The *Act on Protection of Personal Information* of 1995 (2008) applied to public institutions while the *Act on Promotion of Information and Communications Network Utilization and Information Protection, Etc.* of 1999 (2017) applied to the private sector. The PIPA was enacted in 2011 to integrate the two sectors. It emphasizes the right to information privacy by specifying that the right to self-determination of the data subject includes a bundle of rights such as “the right to be informed of the processing of personal information;” “the right to agree or not to the processing of personal information and to the scope of consent;” “the right to request confirmation of personal information processing;” “the right to request access to personal information;” “the right to request the [processor] to suspend, correct, erase and destruct personal information;” and “the right to claim damages that result from personal information processing” (Art 4).

Genetic information is considered “sensitive information” by the PIPA and the BSA. According to the PIPA, sensitive information is defined as “the information that is likely to infringe on the privacy of data provider noticeably, such as ideology, belief, admission to or withdrawal from a trade union or political party, political opinions, health, sexual life, etc.” (Art 23-1). Accordingly, personal medical information is classified as sensitive information (Art 18) and thus requires a high level of protection. It is possible to process sensitive information only when the data processor obtains the consent for such data transfer in a separate consent form from the original consent to the processing of personal information, or where other statutes permit international processing of the data (Art 23-1-1, 2).

Likewise, genetic data included in the medical record are also classified as sensitive information and protected as such, like any other kind of medical information. So, the principle of confidentiality that prohibits the disclosure of personal information to third parties, including family members, without consent also applies to genomic data (MSA Art 19). Personal genetic information is included within the notion of sensitive information even if it is obtained from genetic testing for non-clinical or research purpose (BSA Art 23-1, BSA Enforcement Ordinance Art 18). A person who receives sensitive information from a personal information controller should, in principle, not use it or provide it to a third party (Art 23-1). However, there are some exceptions authorized by the PIPA. For example, public institutions can process personal genome data for the purpose of crime prevention and to provide personal data to foreign governments or international organizations in conformity with international agreements or conventions (Art 18-2). Furthermore, the PIPA allows the controller of sensitive personal information to use or share this information for secondary research purposes only if the data are de-identified (Art 18-2-4). There remains the remote possibility that big data could be re-identified and there is no concrete definition of re-identification in the South Korean regulations (Shin 2018). Current privacy approaches in academic research based on de-identification (PIPA Art 18-2-4) or anonymization (BSA 38-2) are confusing (Chung 2015). For example, genomic research is understood to be exempt from review by IBC when a biobank provides genome data that is re-identifiable only via matching with another biobank, or when the research is not related to a donor’s specific genetic traits (BSA Enforcement Ordinance Art 33). Exemptions from IBC review applicable to de-identified genetic data do not really benefit research participants. They may provide them a false sense of security since the data remain possibly re-identifiable. Neither does this data de-identification also truly benefit longitudinal studies, since follow-up is only possible with identifiable or coded data (Kwon et al. 2016). Accordingly, it seems more important that the information provider agrees with the protection and processing of personal information by genetic testing companies or public institutions rather than attempting to pose an objective judgment on the potential identifiability of such data. A genetic testing institution must provide genetic test results to the legal representative of an individual if requested (BSA Art 52). If, following an investigation, a genetic testing institution is found to have violated Article 52, the Ministry of Health and Welfare can “revoke the designation or registration of a genetic testing institution or a permission granted to an institution” or order it “to completely or partially suspend its operation for a specified period not exceeding one year” (Art 56-1-2). No such investigation or order has been conducted so far.

## Security measures

As the value of a database grows, the need for a robust security system to protect the personal information against loss, theft, data leakages, counterfeit, falsification and damages increases. Recently, accidents involving health data leakage by public agencies such as the NHI Corporation and the HIRA were revealed to South Korean society (Lee 2017). For example, a medical information programming company which provided medical recording services to claim national health insurance or medical care benefits for 7500 hospitals in South Korea illegally extracted patients’ clinical and prescription data (about 750 million files from 44 million individuals) and the company sold the data to a multinational firm (KH 2015). These incidents can negatively influence public trust in the state as a data steward. Accordingly, the duty of providing security safeguards of the personal information processor has been reinforced through detailed provisions in the PIPA (PIPA Enforcement Decree Art 30).

According to the Public Notice of the Standard Measures to Ensure Safety for Personal Information (Standard Measures) (2017), the duty to safeguard implies that the security measures taken correspond to the category of personal information processed and the amount of personal information recorded (Standard Measures Art 3). The processor has the burden of proof to demonstrate that adequate levels of security safeguards were implemented (Standard Measures Art 3). Standards of security safeguards are divided into technical, administrative and physical measures according to the nature of security controls (Standard Measures Art 4). Specifically, administrative measures include management and supervision of the trustee dealing with personal information processing while technical measures include access control of personal information, checking of connection logs, mandatory inspections of uniquely identifying information and security programs; physical measures include provisions to control the import and export from storage facilities and devices and auxiliary storage media containing copies of personal information.

Among personal information, biological information, including genetic information, should be more strictly managed through a password or a personal identification number (PIN) (Standard Measures Art 7-1, 2). The personal information processor must store biometric (including genetic) information and encrypt it when transmitting through an information communication network or transmitting through an auxiliary storage medium.

Regardless of the presence of these security measures, information leakage remains a serious issue in governmental public health databases. The National Health Insurance Corporation imposed disciplinary sanctions on six employees in 2002 (Lee 2007), and 15 more employees have recently received disciplinary sanctions for illegally browsing personal information and data leakages that occurred from 2014 to 2017 (Lee 2017). The previously described incident involving the HIRA service is another case in point.

Another problem is the insufficient level of protection for mobile devices and networks. In some precision medicine initiatives, personal biometric and real-life information of individuals are collected on mobile devices and transmitted to a database. However, the PIPA was designed to promote protection measures such as physical infringement measures, including blocking access to lost or stolen mobile devices, controlling access to personal information using open-wifi networks, and encryption of personal information. While smart phones generate and store individual data related to personalized biology, psychology, behavior and daily environment in real time (Arora et al. 2014), the PIPA does not have regulations on the authentication or data encryption applicable to these devices. Therefore, when someone other than the mobile device owner accesses the mobile data to collect information, uses it without permission or secretly sends inaccurate data, it is difficult to identify the mobile owner’s information in the collection process or in a database. This lack of oversight may cause damage to public trust in databases, data users and researchers due to the risk of inaccurate analysis and results. Inaccurate predictive information could eventually be harmful for research participants, particularly in high-risk populations (Maier et al. 2018; Johnson and Ghlert 2014; Wade et al. 2013).

## International data sharing: compatible processing/adequacy

Automated information processing allows instant access to genomic data crossing borders in terms of geographic space, making it easy and fast to deliver information that is needed by research collaborators worldwide. Therefore, the principle of the PIPA is to establish protection from infringement of personal information that is sent beyond national borders (Art 14). Concrete conditions include the need to obtain the consent from the data subject before transferring personal data to a third party overseas and to respect all applicable requirements set forth by the PIPA (Art 17-3) including the processing of personal information, the safeguard of personal information and guarantees of rights of data subjects.

The main difference between South Korea and the EU’s GDPR is that there is no provision in this country to judge compatible adequacy in international transfers of personal data. There are no specific data sharing rules or distinctions based on the geographical origin of the third party. The BSA also has no concept of compatible adequacy in genomic data sharing. Regardless of whether national or international, once the genome data are consented to be provided to a third party, it can be provided following the decision of the researcher or biobank which intends to conduct the data sharing, and the approval of an ethics committee.

An adequacy assessment is generally required for third countries to process European data. It will be challenging for South Korean privacy law to integrate new European requirements. For instance, there is no clear emphasis on the right to be forgotten and on the right to object automated processing, including profiling, in South Korean law.

Moreover, privacy regulations applicable to data de-identification or anonymization will need to be changed to better align with international data sharing standards (Phillips and Knoppers 2016; Kwon et al. 2016). Pseudonymization is an example of a concept that is not introduced in the PIPA. In Article 4(5) of the GDPR, it is defined as “the processing of personal data in such a manner that they can no longer be attributed to a specific data subject without the use of additional information, with technical and organizational measures to ensure that they are not attributed to an identified or identifiable natural person.”

## Oversight

Although research with de-identified genomic data is considered low-risk research, ethics review for genome data sharing, including cross-border sharing with third parties, is mandated by the BSA Article 10. First, an ethics committee reviews and approves the content of the written consent provided by the research participants according to the legal and ethical requirements for genomic research or biobanks. The participant can indicate personal preference to provide data to a third party, with personal identifying information or not, in the consent form. Then, the biobank approves the data access request through the Distributive Review Committee (DRC) in the National Biobank of Korea under the *Regulation on Operation and Management of the National Biobank of Korea* (2018). For other regional biobanks, alternative ethics committees carry out ethics review (Kim et al. 2017). When an institution or a group submits a plan for the use of human material, the result of the IBC review and relevant documents are sent to the head of the KCDC, the DRC reviews the plan for the use of human material and assesses whether to provide the human material or not (Korea Center for Disease and Prevention 2018).

Internationally, the need to obtain approval to use controlled sensitive data from an access committee can result in time delays and additional costs for researchers (Joly et al. 2012). However, similarly to Canada, there is no provision enabling a one-stop, streamlined ethics or access review process for data sharing in South Korea. This means that, even if multiple institutions engage in collaborative research, it is mandatory to obtain approvals from each institution participating in the initiative.

## Conclusion

In this article, we described the complex South Korean legal and political framework applicable to international genomic data sharing. The landscape is currently undergoing profound changes as diverse stakeholders are currently actively working to update the legal and administrative frameworks for data sharing and governance and common standards are being discussed and established at the local, regional and international levels. These activities contribute to the assessment of the relevance of specific restrictions in national laws and guidelines and to the development of international harmonized principles for responsible data sharing inspired by the latest development from international organizations such as Global Alliance for Genomics and Health (Knoppers 2014).

However, even though the South Korean government is striving to increase the sharing of health and genomic data for research and development, the privacy framework applicable to this information has, until now, developed slowly. Only a few rules are specifically designed for the regulation of collaborative research involving international data sharing. One important issue is that existing Korean regulations have a strong focus on data protection and insufficient consideration on facilitating data usage. This state of affairs reflects public distrust and concerns about large databases due to a series of data misuse incidents that affected Korean society. Furthermore, legislative efforts to facilitate responsible data sharing of genomic and health-related data will need to be implemented. Such regulations applicable to cross-border data transfers will need to achieve a delicate balance between the promotion of data sharing, the improvement of mutual compatibility with western countries, and necessary restrictions to promote better accountability of all stakeholders in the data usage chain.

## References

[CR01] Act on Promotion of Information and Communications Network Utilization and Information Protection, Etc. of 1999 (2017) No. 14839. http://www.law.go.kr/lsInfoP.do?lsiSeq=195040&efYd=20170726#0000. Accessed 20 May 2018

[CR57] Act on the Protection, Use, Etc. of Location Information of 2005 (2017) No. 14840. http://www.law.go.kr/LSW//lsSc.do?tabMe nuId=tab18 &p1=&subMe nu=1&nwYn=1§ion=&tabNo =&query =%EC%9C%84%EC%B9%98%EC%A0%95%EB% B3%B4%EC%9D%98%20%EB%B3%B4%ED%98%B8%20 %EB%B0%8F%20%EC%9D%B4%EC%9A%A9%20 %EB%93%B1%EC%97%90%20%EA%B4%80%ED%95%9C%20- %EB%B2%95%EB%A5%A0#undefined. Accessed 20 May 2018

[CR59] Act on Protection of Personal Information Maintained by Public Institution of 1995 (2008) No. 8871. http://www.law.go.kr/LSW/lsInfoP.do?lsiSeq=84173#0000. Accessed 18 May 2018

[CR1] Allen C, Des Jardins TR, Heider A, Lyman KA, McWilliams L, Rein AL, Schachter AA, Singh R, Sorondo B, Topper J, Turske SA (2014). Data governance and data sharing agreements for community-wide health information exchange: lessons from the beacon communities. EGEMS.

[CR2] Arora S, Yttri J, Nilson W (2014). Privacy and security in mobile health (mHealth) research. Alcohol Res.

[CR3] Bioethics and Safety Act of 2005 (2017) Act No. 15188. http://elaw.klri.re.kr/kor_service/lawView.do?hseq=46341&lang=ENG. Accessed 20 May 2018

[CR4] Borry P, Bentzen HB, Budin-Ljøsne I, Cornel MC, Howard HC, Feeney O, Jackson L (2018). The challenges of the expanded availability of genomic information: an agenda-setting paper. J Community Genet.

[CR5] Bovenberg JA, de Hoogh K, Knoppers BM, Hveem K, Hansell AL (2016). Don’t take it personal: European Union legal aspects of procuring and protecting environmental exposure data in population biobanks through the use of a geo-information systems toolkit. Biopreserv Biobank.

[CR6] Cancer Control Act of 2003 (2017) Act No. 14888. http://elaw.klri.re.kr/kor_service/lawView.do?hseq=45565&lang=ENG. Accessed 20 May 2018

[CR7] Chu M (2017) Government to invest W63 billion into precision medicine. Korea Biomedical Review. http://www.koreabiomed.com/news/articleView.html?idxno=1267. Accessed 12 May 2018

[CR8] Chung Y (2015) De-identification Policy of Personal Information and Tasks on Healthcare Big Data. Health and Social Welfare Forum 227:50–60. https://www.kihasa.re.kr/web/publication/periodical/view.do?menuId=48&tid=38&bid=19&aid=382&ano=5. Accessed 18 May 2018

[CR9] de Montioye YA, Hidalgo CA, Verleysen M, Blondel VD (2013). Unique in the crowd: the privacy bounds of human mobility. Sci Rep.

[CR10] Devaney S (2017) All of Us Research Program. https://www.nichd.nih.gov/sites/default/files/about/advisory/council/archive/201701/Documents/201701_devaney.pdf. Accessed 20 May 2018

[CR11] Drummer TJB, Awadalla P, Boileau C, Craig C, Fortier I, Goel V, Hicks JMT, Jacquemont S, Knoppers BM, Le N, McDonald T, McLaughlin J, Mes-Masson AM, Nuyt AM, Palmer LJ, Parker L, Purdue M, Robson PJ, Spinelli JJ, Thompson D, Vena J, Zawati M, The CPTP Regional Cohort Consortium (2018). The Canadian Partnership for Tomorrow Project: a pan-Canadian platform for research on chronic disease prevention. CMAJ.

[CR12] Gang H (2016) National-level use of health care big data and its policy implications. Health and Social Welfare Forum 238: 55–71. https://www.kihasa.re.kr/english/publications/eng_research/view.do?pageIndex=2&keyField=&key=&menuId=73&tid=34&bid=39&division=&ano=945. Accessed 18 May 2018

[CR13] Global Alliance for Genomics and Health (2014) Framework for responsible sharing of genomic and health-related data. https://www.ga4gh.org/ga4ghtoolkit/regulatoryandethics/framework-for-responsible-sharing-genomic-and-health-related-data/. Accessed 21 June 2018

[CR14] Grady C, Eckstein L, Berkman B, Brock D, Cook-Deegan R, Fullerton SM, Greely H, Hansson MG, Hull S, Kim S, Lo B, Pentz R, Rodriguez L, Weil C, Wilfond BS, Wendler D (2016). Broad consent for research with biological samples: workshop conclusions. Am J Bioeth.

[CR15] Grossman RL, Heath AP, Ferretti V, Varmus HE, Lowy DR, Kibbe WA, Staudt LM (2016). Toward a shared vision for cancer genome data. N Engl J Med.

[CR16] International Bioethics Committee of the UNESCO (2017) Report of the IBC on big data and health. UNESCO. SHS/YES/IBC-24/17/3 REV.2. http://unesdoc.unesco.org/images/0024/002487/248724e.pdf Accessed 4 July 2018

[CR17] Jeong G (2017). Assessment of direct-to-consumer genetic testing policy in Korea based on consumer preference. Public Health Genom.

[CR18] Johnson KJ, Gehlert S (2014). Return of results from genomic sequencing: a policy discussion of secondary findings for cancer predisposition. J Cancer Policy.

[CR19] Joly Y (2018) Dynamic consent: an identity crisis? The 2nd Workshop of the Precision Medicine Project in South Korea **(presentation)**

[CR20] Joly Y, Dove ES, Knoppers BM, Bobrow M, Chalmers D (2012). Data sharing in the post-genomic world: the experience of the International Cancer Genome Consortium (ICGC) Data Access Compliance Office (DACO). PLoS Comput Biol.

[CR21] Kaye J, Whitley EA, David L, Morrison M, Teare H, Melham K (2015). Dynamic consent: a patient interface for twenty-first century research networks. Eur J Hum Genet.

[CR22] KH (2015) Patient records leak. Korea Herald. http://www.koreaherald.com/view.php?ud=20150726000368. Accessed 15 Jun 2018

[CR23] Kim H, Kim S, Hong S, Kim S (2017). Ethical and regulatory considerations on biobanking in the Republic of Korea. Asian Bioeth Rev.

[CR24] Knoppers BM (2014). Framework for responsible sharing of genomic and health-related data. HUGO J.

[CR25] Knoppers BM, Harris JR, Budin-Ljøsne I (2014). A human rights approach to an international code of conduct for genomic and clinical data sharing. Hum Genet.

[CR26] Korea Center for Disease and Prevention (2018) Regulation on the operation and the management of the National Biobank of Korea No. 328. http://www.law.go.kr/LSW/admRulLsInfoP.do?admRulSeq=2100000115950. Accessed 20 June 2018

[CR27] Korea Institute of S & T Evaluation and Planning (2017) 2016 Report of preliminary feasibility study on precision medicine R&D program based on integration of genomics and health-ICT. http://www.kistep.re.kr/c3/sub2_4.jsp?. Accessed 20 June 2018

[CR28] Korea Ministry Health and Welfare (2016) The possibility to manage and store Electronic Medical Records outside the hospital. Public announcement. http://www.mohw.go.kr/react/al/sal0301vw.jsp?PAR_MENU_ID=04&MENU_ID=0403&SEARCHKEY=&SEARCHVALUE=&DATA_GUBUN=&page=3&CONT_SEQ=333740. Accessed 10 May 2018

[CR29] Korea National Institute of Health (2017) Korea Biobank Project. http://www.nih.go.kr/NIH/eng/contents/NihEngContentView.jsp?cid=65660&menuIds=HOME004-MNU2210-MNU2327-MNU2346. Accessed 12 May 2018

[CR30] Kwon SE, Kim DY, Jeong SH, Yoo SY, Kim YH, Park YR, Shim WH, Lee MS, Shin SY, Lee JY, Park JY (2016) A study on promoting legal and political improvement for processing personal information for research purpose: based on public health and clinical researches. Personal Information Protection Commission: Seoul No. 11-1079930-000031-01

[CR31] Lee S (2007) The National Health Insurance Corporation and the National Pension Service seriously leak personal information. Munhwa-Ilbo http://www.munhwa.com/news/view.html?no=20070927010301273030021&w=nv. Accessed 19 Jun 2018

[CR32] Lee I (2017) The National Health Insurance Corporation, In four years, 15 employees punished for personal data leakage. Newsis. http://www.newsis.com/view/?id=NISX20171023_0000125790&cID=10201&pID=10200. Accessed 10 May 2018

[CR61] Maier RM, Zhu Z, Lee SH, Trzaskowski M, Ruderfer DM, Stahl EA, Ripke S, Wray NR, Yang J, Visscher PM, Robinson MR (2018). Improving genetic prediction by leveraging genetic correlations among human diseases and traits. Nature Commun.

[CR33] McMahon AW, Pan GD (2018). Assessing drug safety in children-the role of real-world data. N Engl J Med.

[CR34] Medical Service Act of 1962 (2017) Act No. 14888. http://elaw.klri.re.kr/kor_service/lawView.do?hseq=45565&lang=ENG. Accessed 20 May 2018

[CR35] Minister of the Interior and Safety. Public Notice of Standard Measures to Ensure Safety for Personal Information (2017) Act No. 14888. http://elaw.klri.re.kr/kor_service/lawView.do?hseq=45565&lang=ENG. Accessed 20 May 2018

[CR36] National Research Council (2011) Towards precision medicine: building a knowledge network for biomedical research and a new taxonomy of disease. National Research Council, Washington, DC. https://www.nap.edu/read/13284/chapter/1. Accessed 21 Jun 2018 22536618

[CR37] OECD (2007). OECD principles and guidelines for access to research data from public funding.

[CR38] Office for Government Policy Coordination, Ministry of Interior, Korea Communications Commission, Financial Services Commission, Ministry of Science, ICT and Future Planning, and Ministry of Health and Welfare (2016) Guidelines for the De-identification of Personal Data. Hojeong C&P, Seoul, https://www.privacy.go.kr/eng/news_event_view.do?nttId=7585 Accessed 22 Jun 2018

[CR56] Parker SM (2018). Preventing chronic disease in patients with low health literacy using eHealth and teamwork in primary healthcare: protocol for a cluster randomised controlled trial. BMJ Open.

[CR39] Perkins BA, Caskey CT, Brar P, Dec E, Karow DS, Kahn AM, Hou YCC, Shah N, Boeldt D, Coughlin E, Hands G, Lavrenko V, Yu J, Procko A, Appis J, Dale AM, Guo L, Jönsson TJ, Wittmann BM, Bartha I, Ramakrishnan S, Bernal A, Brewer JB, Brewerton S, Biggs WH, Turpaz Y, Venter JC (2018). Precision medicine screening using whole-genome sequencing and advanced imaging to identify disease risk in adults. Proc Natl Acad Sci USA.

[CR40] Personal Information Protection Act of 2011 (2017) Act No. 14839. http://elaw.klri.re.kr/kor_service/lawView.do?hseq=46731&lang=ENG. Accessed 20 May 2018

[CR41] Phillips M, Knoppers BM (2016). The discombobulation of de-identification. Nat Biotechnol.

[CR42] Phillips M, Dove ES, Knoppers BM (2017). Criminal prohibition of wrongful re-identification: legal solution or minefield for Big Data?. J Bioeth Inq.

[CR43] Ploug T, Holm S (2016). Meta consent—a flexible solution to the problem of secondary use of health data. Bioethics.

[CR44] Rare Disease Management Act of 2016 (2016) Act No. 13667. http://elaw.klri.re.kr/kor_service/lawView.do?hseq=43655&lang=ENG. Accessed 20 May 2018

[CR55] Rathbone AL, Prescott JJ (2017). The use of mobile apps and SMS messaging as physical and mental health interventions: systematic review. J Med Internet Res.

[CR45] Robinson JO, Slashinski MJ, Chiao E, McGuire AL (2015). It depends whose data are being shared: considerations for genomic data sharing policies. J Law Biosci.

[CR47] Rothstein MA (2017). Structural challenges of precision medicine—currents in contemporary bioethics. J Law Med Ethics.

[CR48] Shin S (2018). Issues and solutions of healthcare data de-identification: the case of South Korea. J Korean Med Sci.

[CR49] Shin Y, Choi C, Lee JH, Shin SY (2015). First step to big data research in hospital. MEDINFO 2015: eHealth-enabled Health. IOS Press.

[CR50] Steinsbekk KS, Myskja BK, Solberg B (2013). Broad consent versus dynamic consent in biobank research: is passive participation an ethical problem?. Eur J Hum Genet.

[CR51] Sudlow C, Gallacher J, Allen N, Beral V, Burton P, Danesh J, Downey P, Elliott P, Green J, Landray M, Liu B, Matthews P, Ong G, Pell J, Silman A, Young A, Sprosen T, Peakman T, Collins R (2015). UK Biobank: An open access resource for identifying the causes of a wide range of complex diseases of middle and old age. PLOS Med.

[CR52] UNESCO (2003) International Declaration on Human Genetic Data. UNESCO, Paris, http://www.unesco.org/new/en/social-and-human-sciences/themes/bioethics/human-genetic-data/. Accessed 21 Jun 2018

[CR60] Wade CH, Tarini BA, Wilfond BS (2013). Growing up in the genomic era: implications of whole-genome sequencing for children, families, and pediatric practice. Annu Rev Genomics Hum Genet.

[CR53] Willink A, DuGoff EH (2018). Integrating medical and nonmedical services-the promise and pitfalls of the CHRONIC Care Act. N Engl J Med.

[CR54] You H, Kim S (2015). The rights and criteria of distribution of human biological materials and genetic information. Korean J Med Law.

